# Pharmacovigilance and herbal medicines safety: a cross-sectional study of healthcare professionals’ knowledge, attitudes and practices in selected regions of Tanzania, 2021

**DOI:** 10.1186/s12906-025-05226-w

**Published:** 2025-12-29

**Authors:** Alambo K. Mssusa, Godeliver Kagashe, Sheila Maregesi, Lone Holst

**Affiliations:** 1 Department of Global Public Health and Primary Care, University of Bergen, Bergen, Norway; 2 Muhimbili University of Health and Allied Sciences, School of Pharmacy, Dar Es Salaam, Tanzania; 3 Catholic University of Health and Allied Sciences, School of Pharmacy, Mwanza, Tanzania

**Keywords:** Pharmacovigilance, Phytovigilance, Herbavigilance, Herbal medicine safety, Adverse drug reactions, Healthcare professionals, Tanzania medicines and medical devices authority (TMDA)

## Abstract

**Background:**

The World Health Organization recommends integrating the safety monitoring of herbal medicines into existing pharmacovigilance (PV) systems to avoid overburdening the healthcare systems and national PV centres. However, there are few reports (0.3%) of herbal medicine Adverse Drug Reactions (ADRs) in the global database originating from Africa. An efficient PV system requires the involvement and commitment of healthcare professionals since they are primary contacts in transferring safety information from patients and consumers to national regulatory authorities (NRAs).

**Objectives:**

The aim of this study was to determine the healthcare professionals’ knowledge, attitudes and practices regarding PV and herbal medicine safety in Tanzania.

**Methods:**

An observational, cross-sectional, multicentre study was conducted in five regions of Tanzania, involving healthcare professionals working in pharmacies, hospitals, health centres, dispensaries and accredited drug dispensing outlets. Researcher-administered questionnaires were used.

**Results:**

A total of 380 healthcare professionals participated in the study. The findings showed the majority of the healthcare professionals (65.8%) scored low on knowledge of PV and herbal medicines ADRs (62.9%). Although most respondents (86.8%) expressed favourable attitudes towards ADR reporting, this did not translate into practice, as only a small proportion (22.9%) actively reported ADRs to the NRA, with only 18.4% of the submitted case reports composed of herbal medicines. Most healthcare professionals (70.8%) perceived herbal medicines to be safer than conventional medicines. ADR reports lacked important information for causality assessment and signal identification by the NRA. Reports included all herbal products regardless of their source or origin. Professional background was a significant predictor of knowledge of PV (*p* < 0.001) and herbal medicines ADRs (*p* < 0.001). Pharmacovigilance training and the availability of ADR reporting forms were found to play a significant role in ADR reporting practices (*p* < 0.001).

**Conclusions:**

The study revealed several gaps in the knowledge, attitudes and practices of healthcare professionals in PV and herbal medicines safety monitoring that require several interventions. Targeted efforts are required to promote knowledge and awareness of pharmacovigilance, herbal medicines and their safety to healthcare professionals at all healthcare system levels.

**Supplementary Information:**

The online version contains supplementary material available at 10.1186/s12906-025-05226-w.

## Introduction

Establishing pharmacovigilance (PV) systems to monitor the safety of medical products throughout their lifecycle is important in ensuring consumer safety and reducing the burden of Adverse Drug Reactions (ADRs). The major economic impacts of ADRs include the financial burden on healthcare systems and populations worldwide caused by an increased number of hospital admissions, morbidity, mortality, prolonged hospitalizations and supplementary medical therapeutic interventions [1]. Besides healthcare costs, ADRs also have social impacts on the populations, including impaired quality of life, loss of productivity, disability, impairment and lack of trust in the healthcare system and products [2]. The World Health Organization (WHO) recommends that PV systems should encompass safety monitoring of a scope of products consumed by the population, which includes herbal medicines, traditional and complementary medicines, blood products, biologicals, vaccines, and medical devices [3].

The WHO defines “herbal medicines as including herbs, herbal materials, herbal preparations and finished herbal products that contain, as active ingredients, parts of plants, other plant materials or combinations thereof. In some countries, herbal medicines may traditionally contain natural organic or inorganic active ingredients not derived from plants, such as animal and mineral materials” [4]. The safety monitoring of herbal medicines is a recent addition to the national PV systems in many countries. The WHO emphasized the importance by introducing the guidelines for monitoring the safety of herbal medicines in PV systems in 2004 and a subsequent global survey of its member states on traditional medicines policies and herbal medicines regulation in 2005 [4, 5]. According to the guidance, WHO recommends that herbal medicines safety monitoring not be segregated but rather integrated into the existing countries’ PV systems to avoid overburdening healthcare systems and national PV centres [4].

In many countries in Africa that have introduced herbal medicines safety monitoring, the surveillance relies primarily on a spontaneous reporting system as the main source of safety data based on voluntary submission of ADR cases from consumers and healthcare professionals [6]. Despite its wide usage and advantages, the spontaneous reporting system has been proven to have a problem of underreporting of ADR cases from healthcare professionals [7]. The WHO Programme for International Drug Monitoring (WHO IPDM) global database [8] has included the collection of individual case safety reports (ICSRs) from herbal medicines since 1968; however, the report from the study of van Hunsel et al. (2022) showed the Africa region contributed only 0.3% of the total number of reports [9]. The factors leading to low reporting vary depending on country settings and should therefore be studied to design tailor-made interventions [10].

Healthcare professionals play a critical role in PV systems since they are the primary point of contact for consumers who have experienced ADRs and serve as a link between the regulatory authorities and consumers. When a consumer experiences ADRs from any product, even a self-administered herbal product, they will seek medical counsel from any health facility, regardless of the origin of the product consumed. Healthcare professionals at all healthcare levels have a responsibility to identify the ADRs, manage and counsel the patients and report the ADRs to the NRA [11].

Previous studies conducted among healthcare professionals in Sub-Saharan Africa, South Africa, Ethiopia and Turkey have consistently demonstrated that knowledge, attitudes and practices are critical determinants of pharmacovigilance activities, particularly influencing underreporting [12–15]. Nevertheless, the majority of this research concentrated on PV in conventional medicines, with limited studies that included the safety of herbal medicines [12, 16–18]. This gap highlights the need for studies that specifically examine healthcare professionals’ perspectives on herbal medicine safety within the broader PV framework.

In the global database, among the herbal medicine reports with a known source of origin, 64% originated from healthcare professionals, with physicians/medical doctors leading in reporting a high number of herbal medicine ADR cases [9]. This highlights the critical role of healthcare professionals in generating high-quality safety data and emphasizes the recommendation that they should be equipped with adequate knowledge of PV and herbal medicine safety to ensure the submission of reliable reports [4]. Nevertheless, previous studies have identified barriers to effective PV activities for herbal medicines by healthcare professionals in some settings, including low levels of knowledge, limited awareness of herbal medicine safety, lack of training and inadequate opportunities for continued education [16, 19, 20]. Despite these challenges, few studies have been conducted in Tanzania to determine the knowledge, attitudes and practices of healthcare professionals regarding PV and herbal medicine safety, as well as factors that may directly influence the number of reports shared with the NRA.

In Tanzania, the scope for PV was expanded to include herbal medicine safety monitoring was included when the Tanzania Medicines and Medical Devices Act, Cap 219, came into effect in 2003 [21]. Subsequently, PV regulations were developed in 2018 along with guidelines for monitoring medicine safety [22, 23]. Despite these efforts, the number of herbal medicines ICSRs submitted from Tanzania to the global database is limited, to contribute to signal identification for decision-making [24]. The reasons for underreporting by healthcare professionals and their lack of contribution to the safety reporting of herbal medicines within the PV system in Tanzania, as far as can be ascertained, have not been studied.

In Tanzania, the use of herbal medicines for the management of diseases is widespread across different patient groups. Studies conducted between 2013 and 2014 and again in 2017 reported prevalence rates of 77.1% among individuals with hypertension and 24.4% among hospitalized hypertensive patients, respectively [25, 26]. Similarly, a 2017 study in the Mwanza region found that 23% of pregnant women reported using herbal medicines, while research in the Tabora region between 206 and 2018 documented a prevalence of 61.2% [27, 28]. At the same time, the number of herbal medicines standardized and in pharmaceutical forms formally registered in Tanzania continues to rise, with 58 herbal products now approved by the Tanzania Medicines and Medical Devices Authority (TMDA) [29].

The widespread use of herbal medicines, global safety concerns and limited ICSRs from Tanzania necessitate the need for the NRA to strengthen PV systems that incorporate herbal medicine safety monitoring. A previous assessment of the Tanzanian PV system on herbal medicine safety monitoring revealed that very few reports were submitted by healthcare professionals to TMDA [30]. This highlights persistent gaps, barriers and challenges within the PV system, which require a deeper understanding of healthcare professionals’ perspectives as key stakeholders. Therefore, this study aimed to determine the healthcare professionals’ knowledge, attitudes and practices regarding PV and herbal medicine safety in Tanzania. The study specifically sought to answer the following research questions;

- What is the level of knowledge among healthcare professionals regarding PV and the safety of herbal medicines?
- What are their attitudes toward PV, herbal medicine safety and ADRs reporting?
- What practices do they follow in patient counselling, safety information communication, training, ADRs data collection, documentation and reporting?
- What barriers and challenges hinder the effective participation of healthcare professionals in PV activities?

## Methodology

### Study design

This was an observational cross-sectional multicentre study of healthcare professionals working in public and private health facilities in Tanzania using researcher-administered questionnaires conducted between September and October 2021.

### Study area

Tanzania had about 31 regions with 1,654 retail pharmacies, 14,537 Accredited Drug Dispensing Outlets (ADDOs) and 8,119 health facilities (hospitals, health facilities and dispensaries), with most of the facilities situated in urban areas [31, 32]. There were about 31,724 healthcare professionals who work with patients in the facilities as per the Tanzania Health Profile 2012/2013 [33]. The study was conducted in five regions, selected at random, which were Dar es Salaam, Dodoma, Pwani, Mwanza and Tanga.

### Study population

The study targeted healthcare professionals from diverse cadres including pharmacists, pharmaceutical technicians, pharmaceutical assistants, ADDO dispensers, physicians/medical doctors and nurses. Participants were drawn from pharmacies, ADDOs, hospitals, health centres, and dispensaries across the selected regions, with a focus on those directly involved in managing ADRs.

### Sampling and sample size

Assuming a minimum prevalence of knowledge on herbal medicines of 50% from a previous study [34], a confidence interval of 95% and a margin of error of 5%, a sample size of 380 healthcare professionals was calculated using Raosoft^®^ sample size calculator [35], based on the total population of healthcare professionals.

Due to limited resources and accessibility, convenience sampling was used, and the facilities were randomly selected from the urban areas in the five regions, depending on the accessibility and willingness of healthcare professionals in the facilities to participate. Although convenient sampling has been previously employed in studies and was found to be useful for exploring knowledge, attitudes and practices regarding herbal medicine safety, it is not without some limitations [36, 37]. One potential limitation is the possibility of missing some perspectives from healthcare professionals practising in rural settings. To minimize this, we included a range of healthcare facilities from both public and private sectors, across different levels of care and incorporated professionals from various cadres to capture diverse experiences.

### Data collection

The questionnaires were structured with questions formulated and adapted from published studies and literature [36, 38, 39]. The questionnaires were also tested for reliability by 20 healthcare professionals on knowledge, attitude and practice using Cronbach’s alpha reliability test, which was set to be equal to or above 0.7. The results of the pilot testing were excluded from the study analysis. The validity of the questionnaire’s information was confirmed by the researchers. The questionnaires were translated into the local language (Swahili) as per the local regulations and to make them easier to understand for healthcare professionals at various levels.

A list of health facilities across the five regions was obtained from TMDA, the Pharmacy Council and local government authorities. Once the facilities were identified, healthcare professionals working in pharmacies, ADDOs, and those managing ADRs in hospitals, health centres and dispensaries were approached in person and invited to participate in the study. This was done consecutively until the targeted sample size was achieved. The researcher introduced the study, provided participants with an information sheet, and addressed any questions to ensure understanding of the study and the rights of the participants. The informed consent forms were signed and dated by the participant after agreeing to participate. A trained researcher administered the structured questionnaires through face-to-face interviews with healthcare professionals, and the responses were recorded in the questionnaires (see Additional file 1).

The questionnaires captured details on knowledge, attitude and practice on herbal medicine safety. Details captured included education level, continued professional development courses, knowledge on ADRs, knowledge on reporting requirements, knowledge of PV, knowledge of herbal medicines’ legal requirements and safety, attitude and practice on herbal medicines. Details on medicine names, descriptions of ADRs, and quantity of ICSRs received were also captured through interview and document review of ADR records, package inserts and labelling for products available at the facility. The questionnaire contained closed-ended questions and Likert scale statements. Open-ended questions on herbal medicine names, ADRs description and reasons for not reporting ADRs were also included.

### Data handling and statistical analysis

Quantitative data from the responses to the questionnaires of healthcare professionals were analyzed to quantify the observations. Quantitative data analysis was conducted using the current version of the SPSS Statistics software for Windows, version 29 [40].

Twenty-one questions in three domains were used to assess knowledge: knowledge of PV (4 questions), herbal medicine safety (10 questions) and legal frameworks (5 questions). Each participant’s number of correct responses was calculated and used to assign an overall proficiency score based on the following criteria;

#### Knowledge of PV scores (Maximum score 4)

- Very Good: 4 correct responses.
- Good: 3 correct responses.
- Acceptable: 2 correct responses.
- Poor: Incorrect responses to one or all questions.

#### Knowledge of herbal medicine safety scores (Maximum score 10)

- Very Good: 8–10 correct responses.
- Good: 6–7 correct responses.
- Acceptable: 4–5 correct responses.
- Poor: Incorrect responses to three or more questions.

#### Knowledge of legal framework scores (Maximum score 5)

- Very Good: 5 correct responses.
- Good: 3–4 correct responses.
- Acceptable: 2 correct responses.
- Poor: Incorrect responses to one or all questions.

This categorical proficiency was then converted into a numerical ordinal scale for quantitative analysis, where the possible scores were: 1 = Poor, 2 = Acceptable, 3 = Good, and 4 = Very Good. Data analysis was performed on the scaled data. Descriptive statistics were computed for individual items: The frequency and percentages of participants achieving each proficiency level were calculated for each of the knowledge questions. For overall knowledge in the themes, a composite knowledge score for the entire cohort was calculated as the average of all the assigned numerical scores [1–4].

The twelve predefined statements on the attitudes of healthcare professionals towards herbal medicines and the reporting of ADRs were used in the study. A Likert scale ranging from 1 to 5 was used, with 1 indicating strongly disagree, 2 disagree, 3 neutral (neither agree nor disagree), 4 agree, and 5 strongly agree. An average score of ≥ 3.5 was considered to indicate positive attitudes towards herbal medicines and ADR reporting.

Thirty-two questions on herbal medicine dispensing, herbal medicine safety, and ADR reporting were asked to assess healthcare professionals’ practice. The frequency of PV activities was determined by five choices of “Always”, “Often”, “Sometimes”, “Rarely” and “Never”. Responses of “Yes” and “No” were also used for the ADR reporting practice. Free text was used to record the open-ended questions on types of ADRs, names of medicines and reasons for not reporting ADRs to the regulatory authority.

Frequencies and percentages were used for categorical variables and for continuous variables, descriptive statistics were used to determine frequencies, percentages, means, medians, interquartile range (IQR) and standard deviations. The Likert scale data on attitude and knowledge scores were tested for normality using the Kolmogorov-Smirnov Shapiro-Wilk test and significant results were interpreted that the data was not normally distributed. The types of ADRs were described using the Medical Dictionary for Regulatory Activities (MedDRA) terminology [41].

Cross-tabulations using the Chi-Square test were performed to analyze categorical comparisons between variables such as demographic characteristics, training and ADR forms availability with ADR reporting. Non-parametric tests; The Mann-Whitney U test (for two-group demographic variables) and the Kruskal-Wallis test (for multi-group demographic variables) were used to compare differences in knowledge, attitude and practice scores between groups. Knowledge scores were dichotomized as “adequate” and “inadequate” and attitudes as “positive” and “negative” for binary logistic regression. The multivariate logistic regression analysis was conducted for dichotomous variables and adjusted for demographic variables, PV training and ADR reporting form availability to identify predictors. Significance levels were set at an alpha p value of less than 0.05, and confidence intervals (CIs) were set at 95% for all test parameters.

## Results

A total of 380 healthcare professionals took part in the study. Of these, 50.5% worked in pharmacies, 22.9% in ADDOs, 14.7% in hospitals, and 11.8% in health centres/dispensaries.

### Knowledge

The majority of the healthcare professionals (65.8%) scored low on PV knowledge. In terms of herbal medication ADRs/SEs knowledge, 62.9% scored poorly on all items, while 96.8% scored poorly on three or more questions. The majority of healthcare workers (89.7%) were unaware of the herbal medicine regulations, guidelines and licensed products. The mean total knowledge score on pharmacovigilance and legal framework was 1.6 ± 0.46, while the mean total knowledge score on the Adverse Reactions/side effects of common herbal medicines used in Tanzania was 1.2 ± 0.40. Table 1 summarizes the findings of an evaluation of healthcare professionals’ knowledge of PV, the legal framework of herbal medicine and the safety of selected herbal medicines found in health facilities in Tanzania.

**Table 1:** Healthcare professionals’ knowledge of pharmacovigilance, herbal medicine legal framework and safety of selected herbal medicines

	Distribution of knowledge levels			
Topic/Herbal name	Poor	Acceptable	Good	Very Good
**Knowledge of pharmacovigilance and the legal framework**	%	%	%	%
Pharmacovigilance concept	77.6	14.7	12.4	5.3
Adverse drug reaction concept	68.7	3.7	22.6	15
Importance of ADR reporting	62.6	11.6	25.3	0.5
ADRs prevention	72.6	9.7	16.8	0.8
Institutions regulating herbal medicines in Tanzania	43.4	0.8	24.5	31.3
Institutions regulating traditional medicines in Tanzania	86.8	7.9	5.3	0
Laws and regulations on herbal medicines	96.3	3.2	0.5	0
Guidelines for herbal medicines	96.3	0.3	1.8	1.6
Registered herbal medicines in Tanzania	45	2.1	43.9	8.9
Knowledge of the Adverse Reactions/Side Effects of the common* herbal medicines in Tanzania				
*Panax ginseng *(Ginseng)	80.8	2.4	16.6	0.3
*Glycyrrhiza glabra *(Liquorice)	92.1	0.5	7.4	0
*Azadirachta indica *(Neem tree)	79.2	3.7	17.1	0
*Aloe barbadensis *(Aloe vera)	79.5	6.1	14.5	0
*Gingko biloba *(ginkgo)	89.2	1.6	8.9	0.3
*Hypericum perforatum *(St. John’s Wort)	94.2	0.8	5	0
*Garcinia cambogia *(Malabar tamarind)	95.5	1.3	3.2	0
*E. angustifolia and E.purpurea *(Echinacea)	97.1	0.5	2.4	0
*Oenothera biennis *(Evening primrose)	94.7	0.5	4.7	0
*Moringa oleifera *(Moringa)	89.7	0.8	9.5	0

*Common=Herbal medicines frequently sold in the pharmaceutical outlets in Tanzania

### Attitudes

**Fig. 1:**
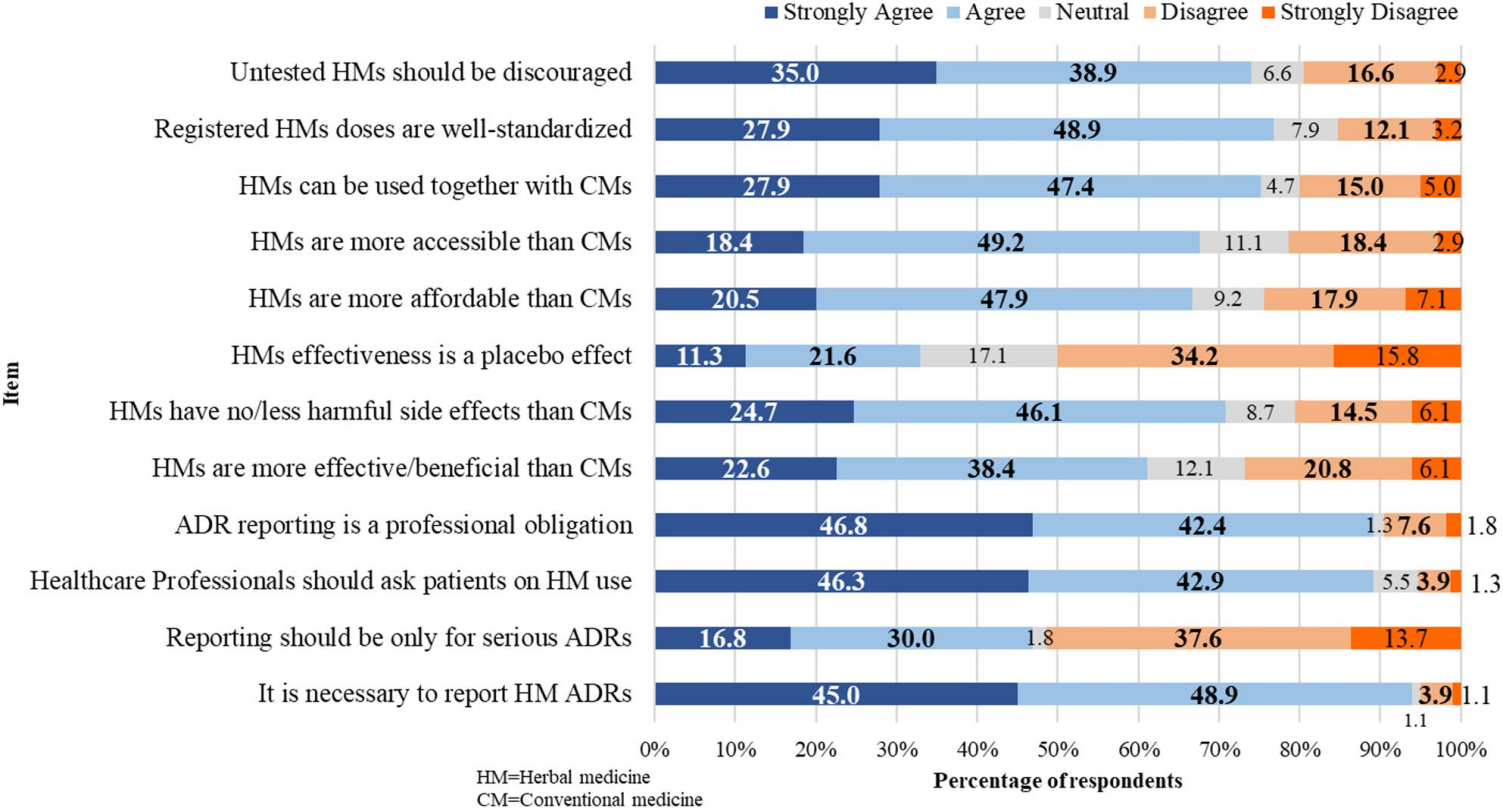
Attitudes on adverse drug reactions (ADRs) reporting and herbal medicines safety (*N* = 380)

The assessment results of attitudes and perceptions towards ADR reporting and herbal medicine safety among participants are described in Fig. 1. The majority of healthcare professionals (86.8%) had positive attitudes towards ADR reporting and 62.9% on herbal medicine therapeutic equity with conventional medicines. The attitudes towards ADR reporting had a total mean score was 3.96 ± 0.06, and towards herbal medicine 3.67 ± 0.54.

**Fig. 2:**
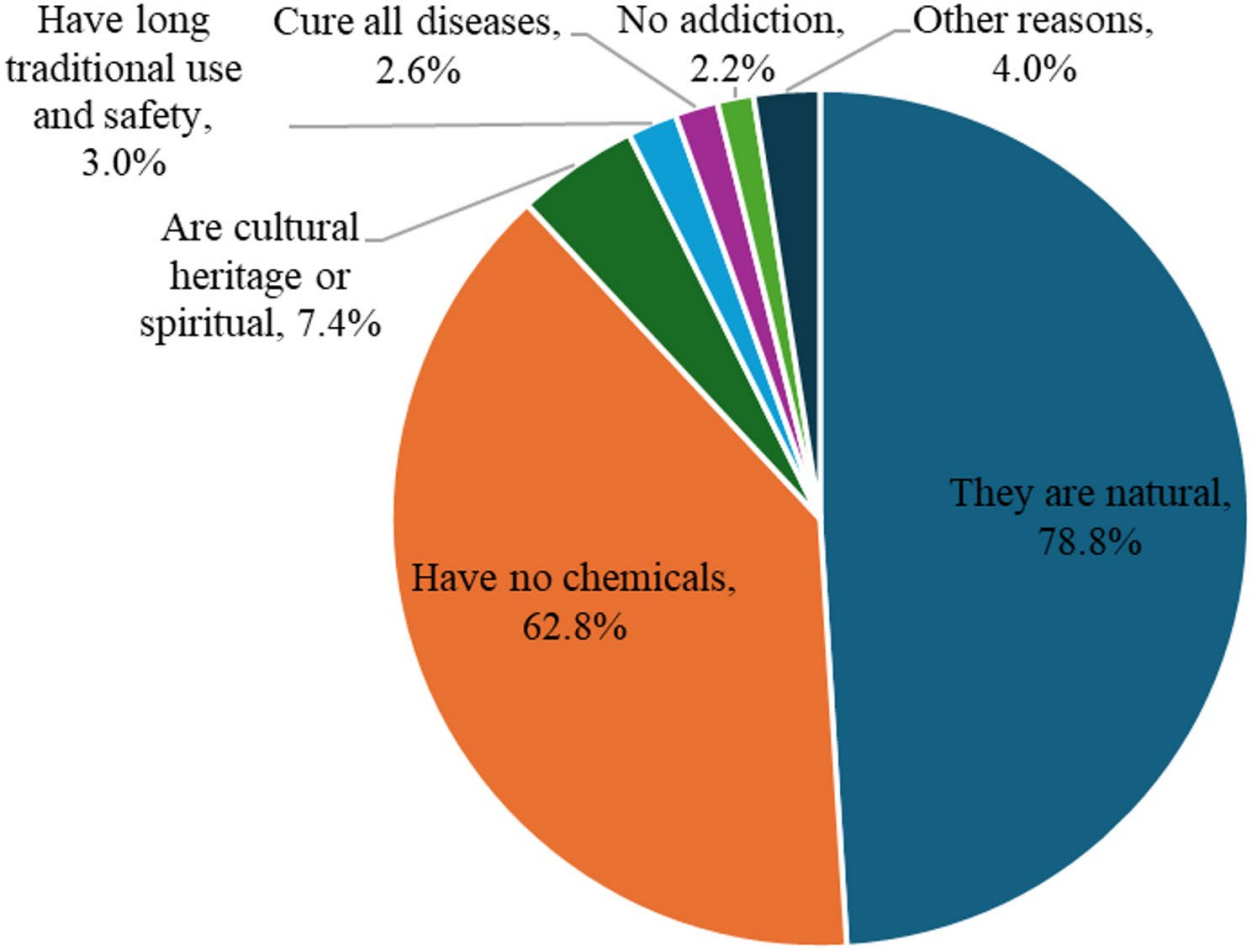
Perceived reasons for the safety of herbal medicines by healthcare professionals

The reasons for perceptions on the safety of herbal medicines compared to conventional medicines among the 269 healthcare professionals who agreed that herbal medicines have no or less harmful SEs than conventional medicines are summarized in Fig. 2.

**Fig. 3:**
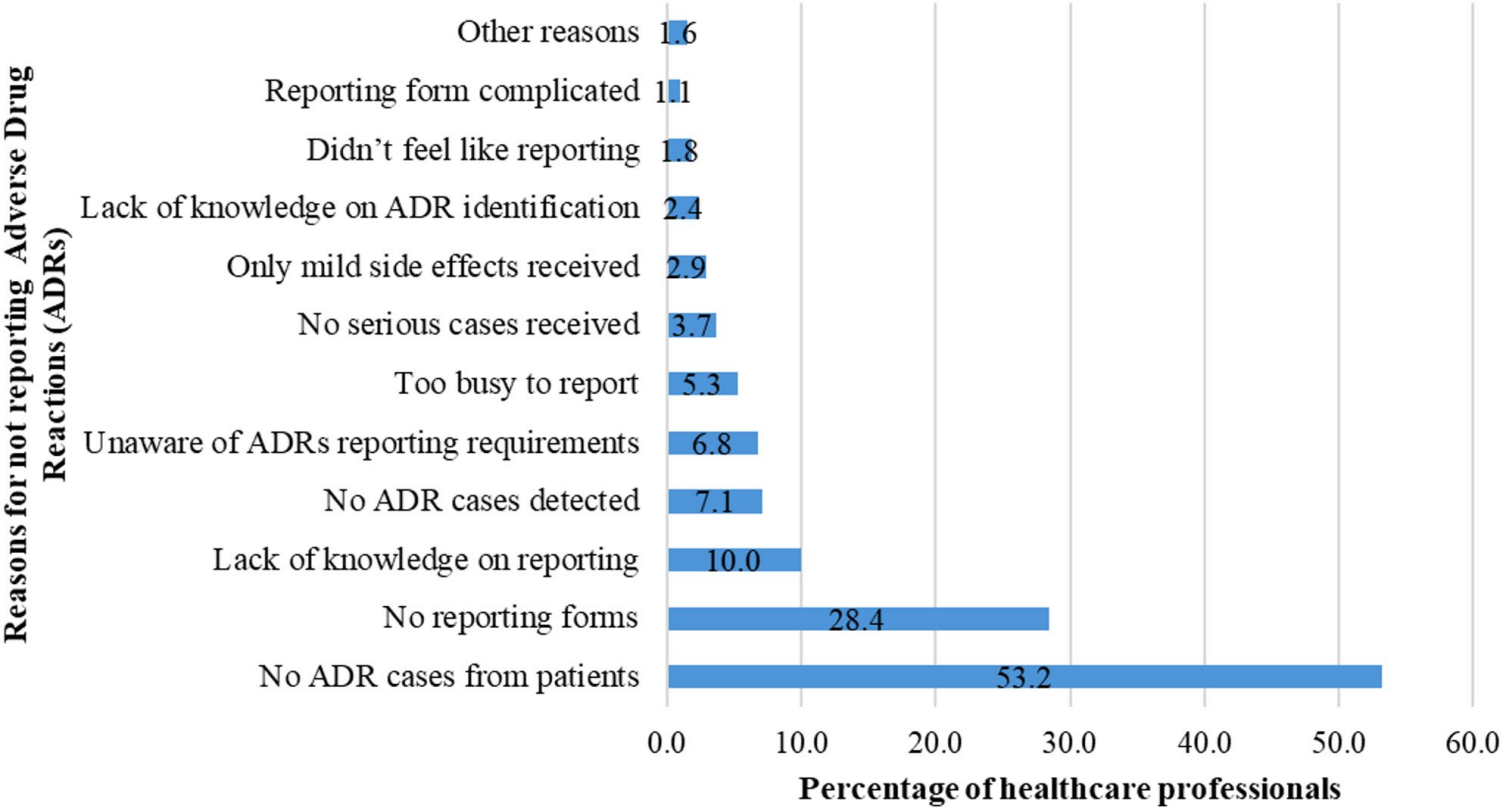
Responses by healthcare professionals on barriers to not reporting adverse drug reactions (ADRs) to the national regulatory authority

Several factors were reported by the healthcare professionals as reasons for not reporting ADRs to the NRA, as shown in Fig. 3. Furthermore, when asked if ADR reporting should be paid for, the majority of healthcare professionals (85.3%) said “No”. In terms of drug interactions, a total of 41.6% out of 370 respondents who were not medical doctors said they did not contact a medical doctor when a drug interaction was encountered, 31% said they did contact a medical doctor, 16.5% said sometimes and 13.5% said they had never encountered any drug interactions.

### Practice

A total of 290 (76.3%) of the healthcare professionals sold herbal medicines in their facilities. The ADR reporting forms were available in only 13.2% of the facilities. The practice of healthcare professionals on medicine safety and reporting is shown in Table 2.

**Table 2:** Healthcare professionals’ practice on medicines safety and reporting

Practice	Always	Often	Sometimes	Rarely	Never
	%	%	%	%	%
Counsel/advise clients regarding ADRs and possible interactions on HMs (*N* = 380)	16.8	8.2	24.2	8.9	41.8
Provide instructions to clients on HMs used (*n* = 290) *	73.4	5.9	11.7	0.3	8.6
Received enquiries on HMs use indications and safety (*N* = 380)	1.3	2.4	8.9	4.5	82.9
	**Yes**		**Sometimes**		**No**
Reported any ADRs to TMDA (*N* = 380)	22.9		-		77.1
Reported ADRs related to herbal medicines to TMDA (*n* = 87) **	18.4				81.6
Report ADRs regularly (*n* = 87)	29.9		44.8		25.3
Used an electronic system for reporting ADRs (*n* = 87)	48.3		-		51.7

*ADR* Adverse Reaction, *HM* Herbal Medicines

*Number of healthcare professionals who sold herbal medicines in their facilities

**Number of healthcare professionals who reported ADRs

Only a few ADR cases involving herbal medicines (6.1%), herbal drug interactions (2.6%), or herbal-herbal interactions (1.3%) were reported to healthcare professionals. The ADR cases received at the facilities ranged from one to three annually. The 23 ADR cases involved 45 Adverse Events (AEs). The most common AEs received are described in Table 3. The healthcare professionals revealed that most of the consumers were unaware of the botanical names and other details of the herbal medicines they consumed.

**Table 3:** Presumed common herbal medicine adverse drug reactions (ADRs) reports received by the healthcare professionals

Adverse Event/Reaction	Frequency	Herbal Product *N* = 380
Abdominal cramps	1	*Aloe barbadensis* (Aloe vera)
Abdominal cramps	1	*Cassia angustifolia* (senna) + *Glycyrrhiza glabr*a (Liquorice) + *Malva verticillata* (cluste mallow) + *Eleutherococcus senticosus* (Siberian ginseng)
Abdominal pain	1	*Aloe barbadensis* (Aloe vera)
Abdominal pain	1	*Ocicum sanctum* (Holy basil*)* + *G.glabra* + *Curcuma longa* (Turmeric) + *Zingiber officinale* (Ginger*)* + *Adhatoda vasica* (Malabar Nut) + *Solanum indicum* (Indian nightshade) + *Inula racemosa* (Indian elecampane*)* + *Piper cubeba* (cubeb*)* + *Terminalia belerica* (Beleric myrobalan) + *A. babadensis* + *Mentha Piperata* (peppermint)
Mouth sores	1	*Adhatoda vasica + Ocicum sanctum + Abbies webbiana* (Indian Silver Fir) + *Glycyrrhiza glabra* + *Curcuma longa* + *Piper longum* (Indian long pepper) + *inula racemosa*
Diarrhoea	1	*Cassia angustifolia + Glycyrrhiza glabra + Malva verticillata + Eleutherococcus senticosus*
Vomiting	1	*Ocicum sanctum + Glycyrrhiza glabra + Curcuma longa + Zingiber officinale + Adhatoda vasica + Solanum indicum + Inula racemosa + Piper cubeba + Terminalia belerica + Aloe babadensis & Mentha piperata*
Rash	7	Herbal medicines (unidentified)
Diarrhoea	5	Herbal medicines (unidentified)
Vomiting	4	Herbal medicines (unidentified)
Nausea	3	Herbal medicines (unidentified)
Abdominal cramps	2	Herbal medicines (unidentified)
Mouth sores	2	Herbal medicines (unidentified)
Headache	2	Herbal medicines (unidentified)

Patients reported a total of nine herbal-drug and four herbal-herbal interactions to healthcare professionals. However, the types of interactions were not documented, and some of the names of the herbal medicines were unidentified.

A total of 74.5% of the participants had access to information regarding herbal medicines from various sources. These sources were: patient information leaflets/product information sheets (63.3%); the internet (35.3%); product labels (9.5%); suppliers (7.1%); books (6%); literature (5.7%); social media (4.2%); healthcare professionals (2.1%); manufacturers (2.1%); experienced experts (2.1%); herbalists (0.7%); and medical representatives (0.7%).

### Training on pharmacovigilance and herbal medicines safety on the knowledge scores performance

Few (13.2%) healthcare professionals were trained in herbal medicines and PV. Details on the types of training received by healthcare professionals are described in Additional File 2. The results of the Pearson’s Chi-square test comparing the differences in knowledge scores between healthcare professionals who received PV training and those who did not are presented in Table 4.

**Table 4:** Comparison of knowledge scores between healthcare professionals trained and not trained in pharmacovigilance (PV)or medicine safety

Knowledge of PV and the legal framework	Knowledge level distribution				
	Trained		Untrained		
	Low score*	High score**	Low score	High score	*p*-value^#^
Pharmacovigilance concept	53.1	46.9	87.6	12.4	< 0.001
ADR concept	46.9	53.1	76.5	23.5	< 0.001
Importance of ADR reporting	38.5	61.5	76.8	23.2	< 0.001
ADRs prevention	62.1	37.9	84.2	15.8	< 0.001
Laws and regulations on herbal medicines	90.0	10.0	97.7	2.3	0.002
Guidelines for herbal medicines	89.9	10.1	98.1	1.9	0.001
Herbal medicines registered in Tanzania	59.1	40.9	43.1	56.9	0.018
Knowledge of adverse reactions/side effects of herbal medicines					
*Glycyrrhiza glabra* (Liquorice)	86.8	13.2	93.9	6.1	0.043
*Aloe barbadensis* (Aloe vera)	73.0	27.0	87.1	12.9	0.005
*Gingko biloba* (Gingko)	80.3	19.7	92.9	7.1	0.001
*Hypericum perforatum* (St. John’s Wort)	89.9	10.1	96.1	3.9	0.032

*Low Poor score, **High score Good and very good scores, *ADR* Adverse drug reaction

^#^Pearson χ2

The majority of healthcare workers who lacked PV training (68.7%) performed poorly in terms of PV knowledge and herbal medicine safety. A highly significant association was found between PV training and PV knowledge (*P* < 0.001). Training in PV also significantly improved knowledge scores regarding laws, regulations and guidelines on herbal medicines (*p* = 0.002, *p* = 0.001 and *p* = 0.018, respectively). Significant results were also found for knowledge of four herbal medicine ADRs/SEs only as described in Table 4 and non-significant results for the remaining 6 types of herbal medicine.

### Demographic characteristics and system factors in ADR reporting practices

Pearson’s chi-square tests were used to evaluate associations between healthcare professionals’ demographic characteristics, pharmacovigilance training, availability of ADR reporting form, and reporting practices for both conventional and herbal medicines as presented in Table 5. Significant differences were observed in reporting practices for healthcare professionals who were trained in PV and who had ADR reporting forms available (*p* < 0.001). Significant gender differences were found in reporting practices for all ADRs, with males reporting more than females (*p* = 0.017). However, for herbal medicines ADRs alone, females reported more than males (*p* = 0.016). Professional background showed that pharmacists had significantly higher ADR reporting rate for all products compared to other professionals (*p* < 0.001). Age and work experience had no significant influence on reporting practice (*p* = 0.773 and *p* = 0.689).

**Table 5:** Association between demographic, system factors and adverse drug reactions (ADRs) reporting practices among healthcare professionals

Demographic characteristic/Item (*N* = 380)	Reported any ADRs to TMDA				Reported ADRs on herbal medicines								
	Yes %	No %	Total (*n*)	*P* value*	Yes %		No %	Total (*n*)			*P* Value*		
Gender													
Male	29.9	70.1	134	**0.017**	7.5		92.5			40			**0.016**
Female	19.1	80.9	246		27.7		72.3			47			
*Profession*													
Pharmacist	47.9	52.1	71	**< 0.001**	11.8		88.2			34			0.171**
Pharmaceutical technician	26.2	73.8	126		15.2		84.8			33			
Pharmaceutical assistant	11.8	88.2	34		50.0		50.0			4			
ADDO dispenser	7.6	92.4	79		33.3		66.7			6			
Physician/Medical Doctor	13.6	86.4	22		33.3		66.7			3			
Nurse	14.6	85.4	48		28.6		71.4			7			
*Availability of ADR reporting forms*													
Forms available	58.0	42.0	50	**< 0.001**	20.7		79.3			29			0.696
No forms	17.6	82.4	330		17.2		82.8			58			
*Trained in pharmacovigilance/medicines safety*													
Trained on PV	50	50	70	**< 0.001**	14.3		85.7			35			0.417
No PV training	16.8	83.2	310										

*ADDO* Accredited Drug Dispensing Outlet

*Pearson χ2

**Fisher Exact Test

### Relationship between variables across demographic characteristics

Since the data on knowledge, attitude and practices did not assume normality, non-parametric tests were used to determine differences between groups. A comparison of knowledge, attitudes and practices on PV and herbal medicine across demographics of healthcare professionals using the Mann-Whitney U Test for two groups (gender) and the Kruskal-Wallis Test for more than two groups was done and the results are shown in Table 6. The results highlighted significant differences between groups in knowledge, attitudes toward ADR reporting, herbal medicine safety and practices among healthcare professionals across sex, education and professional background.

**Table 6:** Comparison of scores on knowledge, attitude and practice on pharmacovigilance (PV) and herbal medicines safety across demographics of healthcare professionals

Demographic Characteristic		Knowledge on PV		Attitudes on ADR Reporting		HM Safety Knowledge		Attitudes on HM		Practices	
*N* = 380	*n*	Median (IQR)	*P* Value	Median (IQR)	*P* Value	Median	*P* Value	Median (IQR)	*P* Value	Median (IQR)	*P* Value
Sex*											
Male	134	1.5(1.0–2.5.0.5)	**< 0.001**	3.8(3.5–4.0.5.0)	**< 0.001**	1.0(1.0–1.2.0.2)	0.99	3.4(3.0–3.9.0.9)	**< 0.001**	2.0(1.3–2.7)	0.22
Female	246	1.0(1.0–1.8.0.8)		4.0(3.75–4.5)		1.0(1.0–1.2.0.2)		3.8(3.3–3.8)		2.0(1.3–2.3)	
Education**											
Certificate	157	1.0(1.0–1.3.0.3)	**< 0.001**	4.0(4.5–4.3)	0.28	1.0(1.0–1.1.0.1)	0.17	3.9(3.5–4.1)	**< 0.001**	2.0(1.0–2.3.0.3)	0.06
Diploma	134	1.25(1.0–2.0)		4.0(3.5–4.5)		1.0(1.0–1.2.0.2)		3.8(3.8–4.1)		2.0(1.3–2.7)	
Bachelor	84	2.0(1.0–3.0)		4.0(3.5–4.3)		1.0(1.0–1.3.0.3)		3.1(3.2–3.5)		2.0(2.0–2.7.0.7)	
Masters	4	2.5(1.4–3.2)		3.5(3.3–3.9)		1.0(1.0–1.4.0.4)		3.9(2.8–3.0.8.0)		3.0(2.3–3.4)	
PhD	1	2.33(*n* = 1)^#^		2.0(*n* = 1)		1.4(*n* = 1)		1.6(*n* = 1)		1.8(*n* = 1)	
Profession**											
Pharmacist	71	2.3(1.3–3.3)	**< 0.001**	4.0(3.5–4.3)	0.16	1.0(1.0–1.4.0.4)	**0.012**	3.1(2.9–3.5)	**< 0.001**	2.0(2.0–3.0)	**0.02**
Pharmaceutical Technician	126	1.5(1.0–2.0)		4.0(3.5–4.5)		1.0(1.0–1.2.0.2)		3.8(3.1–4.1)		2.0(1.3–2.7)	
Pharmaceutical Assistant	34	1.0(1.0–2.5.0.5)		4.0(3.7–4.3)		1.0(1.0–1.0)		3.9(3.7–4.1)		2.0(1.7–2.7)	
ADDO Dispenser	79	1.0(1.0–2.5.0.5)		4.0(3.8–4.5)		1.0(1.0–1.2.0.2)		3.9(3.6–4.3)		2.0(1.3–2.3)	
Physician/Medical Doctor	22	1.3(1.0–1.6.0.6)		4.0(3.7–4.3)		1.0(1.0–1.0)		3.3(3.0–3.8.0.8)		2.0(1.0–2.1.0.1)	
Nurse	48	1.0(1.0–10)		4.0(3.5–4.3)		1.0(1.0–1.0)		3.5(3.0–4.0)		2.0(1.7–2.3)	

*IQR* Interquartile range, *Mann-Whitney U Test, ** Kruskal-Wallis Test* HM *herbal medicine* ADR *Adverse drug reaction* ADDO *Accredited drug dispensing outlet

^#^*n* = 1 only one respondent; Values rounded to one decimal point for easy presentation

### Predictors of knowledge scores

Predictors of obtaining high scores on PV knowledge were determined by logistic regression and it was found that professional background (*p* < 0.001) overall significantly influenced the scores. Pharmaceutical technicians and assistants [AOR = 0.28, 95% CI (0.15–0.52), *p* < 0.001] and doctors, nurses and ADDO dispensers [AOR = 0.09, 95% CI (0.04–0.21), *p* < 0.001] had lower odds of obtaining adequate knowledge scores on PV. Healthcare professionals who did not have specific PV training were also at lower odds of having adequate PV knowledge by 58% [AOR = 0.42, 95% CI (0.22–0.81), *p* = 0.01]. Sex and working experience were not significant predictors of knowledge on PV (*P* = 0.20 and *p* = 0.07) after adjustment and were retained for possible confounding effects.

In terms of knowledge of herbal medicines ADRs/SEs, profession was found to be the only predictor with doctors, nurses and ADDO dispensers having 81% lower odds of adequate knowledge than pharmacists [AOR = 0.19, 95% CI (0.07–0.48), *p* < 0.001]. Gender, age group, experience and PV training were non-significant once the adjustment was done for these factors.

### Predictors of attitudes on herbal medicines and ADR reporting

Factors influencing healthcare professionals’ attitudes towards herbal medicines when compared to conventional medication were determined using adjusted logistic regression. Healthcare professionals having high knowledge scores had reduced odds of favouring herbal medicines on their safety, effectiveness and accessibility by 54% [AOR = 0.46, 95% CI (0.31–0.69), *p* < 0.001]. Education had a significant influence on attitudes (*p* = 0.01) whereby healthcare professionals with Bachelor [AOR = 0.17, 95% CI (0.05–0.51), *p* = 0.002] and Diploma [AOR = 0.37, 95% CI (0.17–0.79), *p* = 0.01] had lower odds of favouring herbal medicines when compared to certificate holders. Levels of Masters and PhD were non-significant due to small samples (*n* = 5, *p* = 0.99). The professional background was found to be overall significant (*p* = 0.024), although pairwise comparison with pharmacists as a reference was non-significant.

Regarding overall attitudes toward ADR reporting, sex remained a significant predictor of positive attitudes, with females having higher odds than males [AOR = 1.69, 95% CI (1.04–2.74), *p* = 0.04]. Knowledge in PV, practice and other demographic variables did not have a significant influence on attitudes, suggesting other systemic or institutional factors might play a role.

### Predictors for ADR reporting practice

Determination of predictors of reporting practice using adjusted logistic regression showed that pharmacovigilance training and availability of ADR reporting forms were significant predictors. Healthcare professionals who had received PV training [AOR = 2.93, 95% CI (1.55–5.56), *p* < 0.001] and the availability of ADR reporting forms in the facility [AOR = 4.36, 95% CI (2.12–8.95), *p* < 0.001] were both strong positive predictors independently associated with significantly higher odds of ADR reporting. Professional background generally was also found to significantly predict ADR reporting (*p* = 0.04) with physicians and medical doctors [AOR = 4.66, 95% CI (1.1–19.8), *p* = 0.04] and ADDO dispensers [AOR = 4.57, 95% CI (1.6–13.4), *p* = 0.006] more likely to report ADRs compared to pharmacists as a reference. In contrast, neither sex (*p* = 0.63) nor work experience (*p* = 0.82) was significantly associated with ADR reporting after controlling for these factors.

## Discussion

Healthcare professionals are vital in ensuring an efficient and functional PV system. They act as key intermediaries in the transfer of safety information from patients or consumers to marketing authorization holders, manufacturers, and national regulatory authorities. The findings of this study, which examined healthcare professionals across all levels of the healthcare system, showed knowledge gaps in PV and ADRs associated with herbal medicines commonly used in Tanzania, varying attitudes regarding herbal medicines and ADR reporting.

The passive surveillance or a spontaneous voluntary reporting system adopted by many countries in Africa requires healthcare professionals at all levels of the healthcare system to possess sufficient knowledge and skills in PV, ADR detection and reporting to enable regulators to identify new signals from the use of health products, including herbal medicines [42]. Evaluation of knowledge of healthcare professionals in this study showed that most of them lacked adequate knowledge of core PV concepts, ADRs, the importance of reporting ADRs, prevention of ADRs, as well as ADRs associated with the herbal medicines that were commonly available in health facilities in Tanzania. Moreover, they demonstrated insufficient knowledge and limited awareness of the legal and regulatory frameworks governing herbal medicine in Tanzania, even though such products were available and dispensed within their respective facilities, suggesting systemic challenges in policy dissemination and professional education within the healthcare sector.

A previous study conducted with pharmacists in Ethiopia showed that the majority were not familiar with the potential side effects of herbal medicine [43]. In contrast, some studies conducted in Saudi Arabia and Jordan showed pharmacists to be knowledgeable about herbal medicine side effects [44, 45]. Lack of knowledge on herbal medicine ADR/side effects by pharmacists could be explained by lack of comprehensive training on herbal medicines pharmacology, complementary and alternative medicine during university courses, lack of continued education on herbal medicines during practice, lack of safety information on many herbal medicines and unavailability/insufficient reference materials.

Studies conducted in Brazil, Jordan and Nigeria found that healthcare professionals’ knowledge on detection, reporting and management of ADRs from health products including herbal medicines is increased by comprehensive training on PV/medicine safety [46–48]. The findings of this study revealed that the vast majority of healthcare professionals lacked training of herbal medicines (86.8%) and PV/medicine safety in general (81.6%). A significant association was found between PV training and knowledge scores (*P* < 0.001) among healthcare professionals. It was observed that healthcare professionals without specific training in PV/medicine safety were significantly less likely to obtain high scores on PV knowledge. A study in Lebanon showed that the professionals who received training in complementary/alternative medicine products in their college studies were more likely to obtain high knowledge scores [49]. The inclusion of PV sessions in all healthcare professional training using a comprehensive modular curriculum recommended by WHO and the International Society of Pharmacovigilance (ISOP) that includes herbal medicine ADRs should be advocated by the countries [50].

In Tanzania, PV content is largely confined to pharmacy curricula and provides limited coverage of herbal medicine safety [30]. To address this gap, regulatory authorities, in partnership with academic institutions, should develop competency-based pre-service and in-service modules for all healthcare cadres. These modules should integrate herbal medicine pharmacology, phytovigilance, nutraceuticals, and complementary/alternative medicine.

Results of the non-parametric analysis to determine differences in knowledge scores across various demographic groups revealed statistically significant differences in knowledge scores on gender, education level, work experience and type of profession, with pharmacists scoring higher than other professionals. However, when adjusted for confounders, the predictors of knowledge on PV were found to be professional background and PV training. When compared to pharmacists, other healthcare professionals (pharmaceutical technicians, assistants, doctors, nurses and ADDO dispensers were less likely to have adequate knowledge of PV. For herbal medicines ADRs/SEs knowledge, doctors, nurses and ADDO dispensers were also less likely to obtain a high score compared to pharmacists. This could be explained by the fact that professions such as pharmacy have incorporated PV into their undergraduate curricula, making them more familiar with the subject. Furthermore, herbal plants and their safety are included in the Pharmacognosy subject in the pharmacy curricula, giving pharmacists a broader understanding of potential herbal medicine toxicities. A study conducted in the United Kingdom found that healthcare professionals lacked the knowledge necessary to effectively advise patients on the use and safety of herbal medicines [51]. Healthcare professionals in countries like China and India are knowledgeable about both conventional and herbal medicines and therefore have advanced PV systems for monitoring the safety of herbal medicines [52, 53]. This shows the importance of advocacy and training tailored to address other professionals who have encountered herbal medicines for the first time during practice, to monitor the safety of these products effectively.

Positive attitudes towards reporting of ADRs to the regulatory authority observed in this study are consistent with the attitudes observed among healthcare professionals in Australia [54]. It is important to probe patients about their herbal medicine consumption to identify and prevent ADRs potentially caused by their interactions with conventional medicines. Patients have been known to use herbal medicines and not declare them to healthcare professionals during consultations, for fear of being reprimanded [55]. A study conducted in the United Kingdom revealed that 63% of the consumers of herbal medicines with conventional medicines would not inform their doctors, and 82% did not inform their pharmacists [56]. Therefore, it is crucial to promote public education on the safety of herbal medicines and their potential interactions, to improve consumers’ reporting of herbal medicines.

The attitude of healthcare professionals on herbal medicine safety in this study, compared to conventional medicines, indicated that the majority perceived herbal medicines as safer or less likely to cause side effects than conventional medicines. The misconception among healthcare professionals that herbal medicines pose minimal safety risks is surprising and inconsistent with established evidence indicating their potential to cause ADRs and toxicity due to their biologically active ingredients, metabolites, contaminants, adulterants and interactions with other herbal products or conventional medicines [57]. This misconception was mainly attributed to the perception that herbal medicines are natural, contain no chemicals and have been culturally used for generations. Comparable attitudes were reported among 61% of pharmacists in a study conducted in Palestine [58]. However, long traditional use or absence of safety data from formal RCTs does not constitute the absence of risks from herbal medicines. These misconceptions about the absence of risks from herbal medicines could be attributed to a lack of knowledge about the safety of herbal products, as was found in the Australian study in 2017, where very few pharmacists recognised ADRs/SEs from herbal products used for weight loss [59]. Similarly, a Serbian study conducted in 2021 revealed that a few healthcare professionals were aware of ADRs caused by herbal-drug interactions [60].

In contrast, a study in Eritrea revealed that the majority of healthcare professionals disagreed with the notion that herbal medicines have fewer side effects compared to conventional medicine [16]. This study found that healthcare professionals with high knowledge scores on PV and those with a higher educational background were significantly less likely to have positive attitudes regarding herbal medicines. In this context, there is a need for countries’ regulatory authorities to implement tailored advocacy interventions to change the attitude and perception of healthcare professionals on herbal medicine safety for an efficient herbal medicines PV system. A study of medical students in Serbia in 2017 showed that equipping medical students with pharmacology education on herbal dietary supplements changed their attitudes and risk perception towards herbal medicines and their ADRs/SEs compared to those without education [61].

This study also revealed that most healthcare professionals believed that herbal medicines could be used concomitantly with conventional medicines. Similarly, a study in Trinidad and Tobago showed that healthcare professionals perceived that such combinations were more effective compared to conventional treatments alone and advocated for integrative medicines [34]. However, caution is warranted as growing evidence highlights the risks of herbal-drug interactions arising from concomitant use of herbal medicines with conventional medicine, particularly through pharmacokinetics and pharmacodynamic mechanisms [62]. A systematic review conducted by Tsai et al. (2000–2010) identified about 1,491 herbal-drug interaction pairs, of which 502 were graded as severe [62]. To minimize these risks, the WHO recommends that herbal medicines should be regulated and undergo a formal registration process to ensure safety information regarding the products is available for post-marketing surveillance [5]. Furthermore, standardization of herbal medicines including identification of active ingredients, quantification of impurities and dose consistency is recommended [63, 64].

In this study, healthcare professionals reported having encountered nine ADRs attributed to herbal-drug interactions and a few involving herbal-herbal combinations, however, inadequate documentation limited the ability to establish definitive causal relationships. These findings highlight the critical need for healthcare professionals to recognize the potential risks associated with herbal medicines to provide informed patient counselling, prevent misdiagnoses, and appropriately manage possible ADRs.

Despite the positive attitudes of healthcare professionals regarding ADR reporting, when it comes to practice, there is still a problem of underreporting at the national level and globally [30]. In this study, the number of ICSRs reported to TMDA was very low compared to the prevalence of herbal medicine use (60–79%) in the population [65], a challenge similarly observed in Lebanon [49]. The knowledge, attitude, beliefs and practice theoretical model expectation is that knowledge influences attitudes and beliefs, which in turn influence practices in PV [66]. However, gaps may occur within this pathway. A study in Saudi Arabia revealed that inadequate knowledge and skills on how and when to report can affect the practices despite healthcare professionals’ positive attitudes [67]. Drawing on the theoretical domains framework (TDF), other factors that can predict intention and behaviour of healthcare professionals on ADR reporting were found to be fear liability, time constraints, unsuitable reporting systems, unclear responsibilities, inadequate teamwork and lack of supportive organisational culture [68–70]. These findings highlight the need to bridge the knowledge, attitudes, beliefs and practices gaps among healthcare professionals through capacity-building interventions, targeted training and system-level improvements.

Several factors were reported by the healthcare professionals that may explain the low ADR reporting rate observed in this study. One of the major factors was patients’ failure to report ADRs, which could be explained by patients’ limited understanding of the potential ADRs and interactions associated with herbal medicines, as observed in a study from Germany [71]. Even when patients report such reactions, healthcare professionals may fail to capture them due to inadequate expertise in detection and reporting. Furthermore, most healthcare professionals, including pharmacists, reported not receiving patient queries on herbal medicine safety. This necessitates the importance of strengthening patient awareness and provider capacity in herbal pharmacovigilance.

This study also found that about half of the healthcare professionals had rarely or never provided any advice or counselling on herbal products, ADRs and possible interactions to their patients. A study by Semple et al. conducted in Australia in 2004 revealed that among the barriers to providing information to consumers on complementary products were inadequate training, inaccessibility to information, time constraints and lack of confidence in information on ADRs/SEs and interactions of the products [72]. Other studies conducted in Singapore and the USA in 2003 also reported that Pharmacists’ lack of knowledge and skills on herbal products and inaccessibility to safety information hindered adequate counselling with consumers [73, 74]. This contrasts with findings from a Palestinian study, where the majority of pharmacists actively counselled patients and received enquiries related to herbal product safety [36].

The availability of ADR reporting forms, whether electronically or in paper-based formats, has been identified as one of the critical interventions to reduce underreporting in countries [75]. In African settings, lack of access to the reporting forms has been documented as a key obstacle to effective ADR reporting for all medical products [76], as also revealed in this study. Furthermore, results showed that the availability of reporting forms significantly increased the submission rates overall (*p* < 0.01) for all products and was a key predictor. These findings highlight the need to complement ADR reporting form accessibility to all healthcare professionals with specific educational efforts on the use of the reporting forms for herbal medicines. Since nearly half of the healthcare professionals who submitted ICSRs did not use the electronic reporting system, the TMDA should promote the simultaneous use of electronic mobile applications such as VigiMobile^®^ [77] alongside paper-based forms to simplify the reporting process and mitigate challenges related to the unavailability of paper forms.

The herbal medicines ADRs reported to be received by healthcare professionals in this study were mainly for gastrointestinal disorders such as abdominal cramps/pain, diarrhoea and vomiting. Similar patterns of gastrointestinal adverse effects related to herbal products have been documented in studies conducted in Saudi Arabia and the Netherlands [78, 79]. However, in this study the quality of the information that was received by healthcare professionals from consumers was notably poor and missed essential details required for effective ADR management, reporting and causality assessment. In most cases in the study, the herbal products could not be identified by names, formulation, amount, frequency and doses used. Furthermore, the consumers reported ADRs from all herbal medicines regardless of their origin and source. These findings underscore the need for a centralized PV hub dedicated to reporting and monitoring all herbal, complementary and traditional medicine products for the integration of herbal medicines into the routine PV system to be effective. This should be supported by a streamlined regulatory framework that mandates registration/listing, clear labelling and standardized documentation of all the products being used by herbal medicine practitioners for proper product identification and robust causality assessments.

Other predictors of the number of ICSRs submitted to the national regulatory authority, as determined through adjusted regression analysis revealed that physicians/medical doctors, clinical officers and ADDO dispensers were approximately five times more likely to report ADRs compared to pharmacists as a reference. This could be explained by the direct exposure of these frontline healthcare workers to patients and consumers as they encounter ADRs. In the Netherlands, herbal medicines ICSRs submitted by healthcare professionals were more from physicians, followed by pharmacists [79]. This emphasizes the need to incorporate PV training across all levels of healthcare professionals’ courses and ensure accessibility to the reporting tools as one of the key interventions to reduce underreporting and strengthen PV systems.

### Strengths and limitations

The major strength of this study is its inclusion of healthcare professionals from various disciplines and from both public and private health facilities across multiple regions and therefore capturing experience in diverse healthcare settings. Additionally, the use of face-to-face interviews with the researcher-administered questionnaires enabled good sample size retention and collection of quality information from the participants compared to self-administered tools. However, the study had some limitations. Convenience sampling was employed in urban and semi-urban areas, which may not have captured the perspectives of some healthcare professionals working in rural settings. To mitigate this, we included multiple healthcare facilities from private and public sectors, at different levels and healthcare professionals from different cadres to obtain diverse experiences. Additionally, since some questions relied on participant recall, there is a possibility of recall bias, which may have influenced the accuracy of the responses provided.

## Conclusion

The study revealed a significant gap in knowledge, attitudes and practices regarding PV and herbal medicine safety among all healthcare professionals in Tanzania which contributed to critical underreporting and poor data quality of ADR reports. Several barriers were identified, including insufficient knowledge and training on PV and herbal medicine safety, lack of access to reporting forms, consumers not reporting, lack of skills on ADR detection, and limited knowledge on reporting requirements. For the pharmacovigilance system to function efficiently, targeted strategies are urgently needed to strengthen knowledge and awareness of PV, herbal medicine safety monitoring among healthcare professionals at all healthcare system levels.

Policymakers and decision-makers should prioritize specialized training to equip healthcare professionals with the competencies necessary for the effective detection, collection, reporting, evaluation and communication of risks associated with herbal medicines. Curricula integrating PV and the safety of herbal medicines should be implemented in all training programmes for healthcare professionals, with modules tailored to different cadres. Future implementation research should evaluate the effectiveness of such training on reporting behaviour and data quality, and pilot targeted capacity-building interventions before scaling up nationally. Furthermore, guidelines, tools, and an official herbal medicine register (similar to a national formulary) that including scientific names, indications, side effects, interactions, precautions and contraindications should be developed and distributed to healthcare professionals for reference.

## Supplementary Information

Additional file 1. Questionnaire for healthcare professionals on herbal medicines safety.

Additional file 2. Healthcare professionals training in herbal medicines and pharmacovigilance.

## Data Availability

The datasets generated and/or analyzed in this study are available from the corresponding author on reasonable request. In the event the data is to be released to the public, all necessary procedures will be adhered to ensure the privacy and confidentiality of participants, including data anonymizations and additional ethical and institutional approvals.

## Abbreviations

ADDO: Accredited Drug Dispensing Outlet
AE: Adverse Event
ADR: Adverse Drug Reaction
AOR: Adjusted Odds Ratio
CI: Confidence interval
GBT: Global Benchmarking Tool
HM: Healthcare Professional
ICSRs: Individual Case Safety Reports
IQR: Interquartile range
IPDM: WHO Programme for International Drug Monitoring
NRA: National Regulatory Authority
PV: Pharmacovigilance
SE: Side effect
TMDA: Tanzania Medicines and Medical Devices Authority
UMC: Uppsala Monitoring Centre
WHO: World Health Organization
