# Supplementary material for: Pharmacovigilance and herbal medicines safety: a cross-sectional study of healthcare professionals’ knowledge, attitudes and practices in selected regions of Tanzania, 2021
Source: BMC Complement Med Ther. 2025 Dec 29;26:37. doi: 10.1186/s12906-025-05226-w (PMC12860113; doi:10.1186/s12906-025-05226-w)
Supplement: Supplementary file 1 — Additional file 1. Questionnaire for healthcare professionals on herbal medicines safety. [file 12906_2025_5226_MOESM1_ESM.docx]

**QUESTIONNAIRE FOR HEALTHCARE PROFESSIONALS ON HERBAL MEDICINES SAFETY**

| **Healthcare professional Details** | |
| --- | --- |
| **Workplace (Name of the Facility):** | **Place of Employment:**   1. Private 2. Public 3. NGO   Pharmacy□ ADDO □ Hospital□ Dispensary□  Health Centre□ |
| **Physical Address:** |  |
| **Sex:** 1.Male  2.Female | **Age (years):**  **Birth date** *(If known): Dd/Mm/Yyyy* |
| **Education:**   1. Diploma 2. Certificate 3. Bachelor 4. Masters 5. PhD 6. Other   **Country of training**   1. Tanzania 2. Outside Tanzania (Specify _____________) 3. Both   **Profession**   1. Pharmacist 2. Pharmaceutical Technician 3. Pharmaceutical Assistant 4. ADDO Dispenser 5. Physician/medical doctor 6. Clinical Officer 7. Nurse | **Work Experience:**………………… *(Years, months, Days)*  **Type of experience**(Tick all that apply)   1. □ Community 2. □Clinical 3. □Sales/Marketing 4. □ Industrial 5. □Regulatory 6. □Academia 7. □Other………………………………………………………   **Experience in Dispensing of herbal Medicines**  ………………………….. (Years, months, Days) |
| **Herbal Medicine and Pharmacovigilance Practice** | |
| 1. Do you sell herbal medicines in your facility? 2. Yes b) No c) Not Applicable 3. If yes, which type of herbal Medicines do you have in your facility?   …………………………………………………………………..  …………………………………………………………………..  ………………………………………………………………….  ………………………………………………………………….  ………………………………………………………………….  …………………………………………………………………  ………………………………………………………………..   1. Which categories of herbal medicines do you sell in your facility? *(Tick all that apply)* 2. Prescription medicines□ 3. Pharmacy only medicines □ | 1. Over the counter medicines□ 2. General sales□ 3. Self-medication only□ 4. Dietary supplements □ 5. Health food □ 6. Functional food □ 7. Others □ specify______________________ 8. Not Applicable 9. Which are the most dispensed herbal products in your facility (top 5)?   a.________________________________  b.________________________________  c.________________________________  d.________________________________  e.________________________________  f. Not Applicable |
| 1. Do you receive enquiries on herbal medicines use, indications and safety? 2. Always 3. Often 4. Sometimes 5. Never 6. Rarely 7. Which herbal medicines do you receive enquiries on?   _______________________________________  _______________________________________  _______________________________________   1. Do you counsel/advice your clients regarding adverse drug reactions and possible interactions on herbal medicines? 2. Always 3. Often 4. Sometimes 5. Never 6. Rarely 7. Which herbal medicines do you counsel/advice on ADRs and possible interaction?   ________________________________________  ________________________________________  ________________________________________   1. Have you ever received cases of adverse drug reactions or side effects from herbal medicines? 2. Yes 3. No 4. If yes, How many cases of herbal medicines ADRs do you receive per month or per year?   ………………………………………………………..(no. of cases)   1. Which types of herbal products were implicated in causing the reactions?   ……………………………………………………………………………………  ……………………………………………………………………………………..  …………………………Describe…………………………………………………………..   1. How many cases have you received in the past five years?   ………………………………………………………..(no. of cases)   1. Are there ADR reporting forms in your facility?   □Yes □No   1. Which groups of population mostly use herbal products in your facility? *(Tick all that apply)* 2. □Cancer patients 3. □HIV Clients 4. □Malaria patients 5. □Weight- Loss clients 6. □Pregnant/ Breastfeeding women 7. □Hospitalized patients 8. Others……………………………………. 9. Which age group does your herbal medicines clients belong to? *(Tick all that apply)* 10. Infants and Children 11. Adolescents and teens 12. Adults 13. Old and Elderly | 1. Do you provide instructions to your clients on herbal medicines use? 2. Always 3. Often 4. Sometimes 5. Never 6. Rarely 7. Have you ever received cases of herbal drug to herbal drug interactions?   □Yes □No   1. If yes which herbal products interacted?   a.________________with_______________  b.________________with_______________  c.________________with_______________   1. Have you ever received cases of herbal drug interactions with conventional medicines?   □Yes □No   1. If yes which herbal products interacted with conventional medicines?   a.________________with_______________  b.________________with_______________  c.________________with_______________   1. Where do you get access to herbal medication   Information?   1. …………………………. 2. …………………………. 3. ………………………….   **ADR Reporting Practice**   1. Have you ever reported any ADRs to TMDA?   □Yes □No   1. Do you report ADRs regularly?   □Yes □Sometimes □No   1. Have you ever reported ADRs related to herbal medicines to Tanzania Medicines and Medical Devices Authority (TMDA)?   □Yes □No   1. Have you ever used electronic system for reporting ADRs? □Yes □No 2. Do you contact a Medical Doctor when you encounter a drug interaction?   □Yes □Sometimes □No □Not Applicable |

| **Attitude/Perception towards herbal medicines** | **Attitude/perception towards reporting Adverse Drug Reactions (ADRs)** |
| --- | --- |
| Please state your agreement with the following statements;   1. Herbal medicines are more effective/Beneficial than conventional medicines   □Strongly agree □Agree □Neither agree nor disagree □Disagree □strongly Disagree □Don’t know   1. Herbal medicines have no or less harmful side effects than conventional medicines   □Strongly agree □Agree □Neither agree nor disagree □Disagree □strongly Disagree □Don’t know   1. If you agree, why do you think that they are safer?   □ No chemicals □Natural □ Cures all diseases  □ Do not expire □ Cultural heritage/Spiritual □ No addiction □Other, specify  …………………………………………………………………   1. The effectiveness of herbal medicines is due to a placebo effect   □Strongly agree □Agree □Neither agree nor disagree □Disagree □strongly Disagree □Don’t know   1. Herbal medicines are more affordable than conventional medicines   Strongly agree □Agree □Neither agree nor disagree □Disagree □strongly Disagree □Don’t know   1. Herbal medicines are more accessible than conventional medicines   □Strongly agree □Agree □Neither agree nor disagree □Disagree □strongly Disagree □Don’t know   1. Herbal medicines can be used together with conventional medicines   □Strongly agree □Agree □Neither agree nor disagree □Disagree □strongly Disagree □Don’t know | 1. Doses of registered herbal medicines in Tanzania are well standardized   □Strongly agree □Agree □Neither agree nor disagree □Disagree □strongly Disagree □Don’t know   1. Herbal medicines that have not been tested scientifically should be discouraged   □Strongly agree □Agree □Neither agree nor disagree □Disagree □strongly Disagree □Don’t know   1. It is necessary to report ADRs/side effects from herbal medicines   □Strongly agree □Agree □Neither agree nor disagree □Disagree □strongly Disagree □Don’t know   1. Reporting of ADRs should be only for serious reactions   □Strongly agree □Agree □Neither agree nor disagree □Disagree □strongly Disagree □Don’t know   1. Pharmacists and medical Doctors should ask the patients if they are using herbal medicines during consultations   □Strongly agree □Agree □Neither agree nor disagree □Disagree □strongly Disagree □Don’t know   1. Reporting of ADRs is a professional obligation/duty   □Strongly agree □Agree □Neither agree nor disagree □Disagree □strongly Disagree □Don’t know   1. In case you have never reported ADRs, what are the reasons?   ____________________________________  ____________________________________  ____________________________________   1. Do you think reporting ADRs should be paid for?   □Yes □No |
| **Knowledge on pharmacovigilance and herbal Medicines Safety** | |
| 1. Do you have training on herbal medication?   □Yes □No   1. What type of training on herbal medicines do you have? Duration?   Type……………………Duration …………………………   1. What is Pharmacovigilance?   ………………………………………………………………………..  Score: □Poor □Acceptable □Good □Very Good   1. What is an Adverse Drug Reaction?   ...........................................................................  Score: □Poor □Acceptable □Good □Very Good   1. Do you have any training in Pharmacovigilance/medicines safety?   □Yes □No   1. What type of training on Pharmacovigilance/ medicines safety do you have? Duration?   Type……………………Duration …………………………   1. Do you think reporting ADRs ensures patient’s safety?   □Yes □No   1. If yes, How?   …………………………………………………………………………..  ……………………………………………………………………………  …………………………………………………………………………..  Score: □Poor □Acceptable □Good □Very Good   1. Can ADRs be prevented?   □Yes □No   1. If yes, How?   …………………………………………………………………………..  ……………………………………………………………………………  …………………………………………………………………………..  Score: □Poor □Acceptable □Good □Very Good   1. Who regulates herbal medicines in Tanzania?   ………………………………………………………………………..  Score: □Poor □Acceptable □Good □Very Good   1. Who regulates traditional medicines in Tanzania?   ………………………………………………………………………..  Score: □Poor □Acceptable □Good □Very Good | 1. Which laws and regulations on herbal Medicines do you know?   ………………………………………………………………………..  ………………………………………………………………………..  Score: □Poor □Acceptable □Good □Very Good   1. Which guidelines of herbal medicines do you know?   ………………………………………………………………………..  Score: □Poor □Acceptable □Good □Very Good   1. Which herbal medicines have been registered in Tanzania?   ……………………………………………………………………  ……………………………………………………………………  …………………………………………………………………...  Score: □Poor □Acceptable □Good □Very Good   1. Do you know any ADRs/Side effects caused by Ginseng?   …………………………………………………………………  ……………………………………………………………………  Score: □Poor □Acceptable □Good □Very Good   1. Do you know any ADRs/Side effects caused by Glycyrhiza glabra (Liquorice)?   ……………………………………………………………………  ……………………………………………………………………  Score: □Poor □Acceptable □Good □Very Good   1. Do you know any ADRs/Side effects caused by Azadirachta indica (mwarobaini)?   ……………………………………………………………………  ……………………………………………………………………  Score: □Poor □Acceptable □Good □Very Good   1. Do you know any ADRs/Side effects caused caused by Aloe vera )?   ……………………………………………………………………  ……………………………………………………………………  Score: □Poor □Acceptable □Good □Very Good   1. Do you know any ADRs/Side effects caused by Gingko Biloba?   ……………………………………………………………………  ……………………………………………………………………  Score: □Poor □Acceptable □Good □Very Good   1. Do you know any ADRs/Side effects caused by St.John’s Wort?   ……………………………………………………………………  ……………………………………………………………………  Score: □Poor □Acceptable □Good □Very Good |
| 1. Do you know any ADRs/Side effects caused by Garcinia cambogia?   ……………………………………………………………………  ……………………………………………………………………  Score: □Poor □Acceptable □Good □Very Good   1. Do you know any ADRs/Side effects caused by Echinacea?   ……………………………………………………………………  ……………………………………………………………………  Score: □Poor □Acceptable □Good □Very Good | 1. Do you know any ADRs/Side effects caused by evening primrose?   ……………………………………………………………………  ……………………………………………………………………  Score: □Poor □Acceptable □Good □Very Good   1. Do you know any ADRs/Side effects caused by *Moringa Oleifera* (mlonge)?   ……………………………………………………………………  ……………………………………………………………………  Score: □Poor □Acceptable □Good □Very Good |

***For Official use of Interviewer***

Please check the types of herbal medicines in stock at the facility and list their common names and brand names (If applicable)

|  |  |
| --- | --- |
|  |  |
|  |  |
|  |  |
|  |  |
|  |  |
|  |  |
|  |  |

Please check if there is an ADR register and record the number of ADRs reported.

|  |
| --- |

Please check if there are any reference materials for herbal medicines and pharmacovigilance available at the facility

|  |  |
| --- | --- |
|  |  |
|  |  |
|  |  |

Name of the Interviewer:

|  |
| --- |

Date of the Interview:

|  |
| --- |

**Definition of terms**

**Herbal Medicine**

A plant-derived material or preparation with therapeutic or other human health benefits which contains either raw or processed ingredients from one or more plants. In some traditions materials of inorganic or animal origin may also be present (WHO 2000).

**Prescription medicines**

Herbal medicines/drugs that can only be purchased with a Prescription i.e. a physician’s order)

**Over the counter medicines**

Herbal medicines/drugs that can be purchased without a prescription from a physician

**Self-medication only**

Medicines/drugs permitted or self-medication purposes only

**Dietary supplements**

A dietary supplement is a substance which contains, for instance, a vitamin, a mineral, a herb or other botanical or an amino acid. A dietary supplement may be intended to increase the total daily intake of a

concentrate, metabolite, constituent, extract or combination of these ingredients

**Health food**

Health foods could be products that are presented with specific Health claims and therefore regulated differently from other foods

**Functional foods**

Like health foods, functional foods may be products which are offered with specified health claims and therefore regulated differently from other foods
