# Supplementary material for: Pharmacovigilance and herbal medicines safety: a cross-sectional study of healthcare professionals’ knowledge, attitudes and practices in selected regions of Tanzania, 2021
Source: BMC Complement Med Ther. 2025 Dec 29;26:37. doi: 10.1186/s12906-025-05226-w (PMC12860113; doi:10.1186/s12906-025-05226-w)
Supplement: Supplementary file 2 — Additional file 2. Healthcare professionals training in herbal medicines and pharmacovigilance. [file 12906_2025_5226_MOESM2_ESM.docx]

**Additional File 2: Healthcare professionals training in herbal medicines and pharmacovigilance**

| **Training on herbal medications** **(N=380)** | **n (%)** |
| --- | --- |
| HCPs with training on herbal medication | 50 (13.2) |
| ***Herbal medicine training type (n=50)*** |  |
| Pharmacognosy | 40 (80.0) |
| Specific product training by the supplier | 7 (14.0) |
| Botany | 2 (4.0) |
| Phytochemistry | 1 (2.0) |
| **Training on Pharmacovigilance/ medicines safety** |  |
| HCPs with training in PV | 70 (18.4) |
| ***PV training type*** |  |
| College/University curriculum | 27 (7.1) |
| PV short courses (physical attendance) | 22 (5.9) |
| ADDO training | 14 (3.7) |
| Online PV short course | 7 (1.9) |
| *ADDO=Accredited Drug Dispensing Outlet; PV=pharmacovigilance; HCP= healthcare professional* | |
